# Editorial: Supporting pediatric drug development: from basic research to clinical studies and technological advancements

**DOI:** 10.3389/fped.2024.1477420

**Published:** 2024-08-21

**Authors:** Nikolas Dietis, Karel Allegaert, Elke Smits, Adriana Ceci

**Affiliations:** ^1^ Medical School, University of Cyprus, Nicosia, Cyprus; ^2^ Department of Pharmaceutical and Pharmacological Sciences, KU Leuven, Leuven, Belgium; ^3^ Department of Development and Regeneration, KU Leuven, Leuven, Belgium; ^4^ Department of Hospital Pharmacy, Erasmus Medical Center, Rotterdam, Netherlands; ^5^ Clinical Research Center, Antwerp University Hospital, University of Antwerp, Antwerp, Belgium; ^6^ Department of Research, Fondazione per la Ricerca Farmacologica ‘Gianni Benzi’ Onlus, Bari, Italy

**Keywords:** pediatric drug development, children, pediatric treatment, EPTRI, preclincical pediatric research

**Editorial on the Research Topic**
Supporting pediatric drug development: from basic research to clinical studies and technological advancements

The shortage or absence of pediatric medicines, along with the lack of specific labeled guidance for their use, remains a significant issue worldwide. Despite existing legal frameworks and incentives, children continue to lack appropriate therapeutic options due to the challenges inherent in pediatric drug development (1). Addressing these challenges requires a comprehensive and integrated approach that encourages collaboration and innovation in pediatric drug development, focusing on age-appropriate formulations, novel biomarkers, and advanced technologies to ensure that children have access to safe and effective treatments.

This Research Topic is introduced and supported by the European Pediatric Translational Research Infrastructure (EPTRI), a European distributed research infrastructure initiated by EU funding (EU-EPTRI-ID n.777554) and established as a non-profit research organisation incorporated in the form of an Association Internationale Sans But Lucrative (AISBL) governed by Belgian law, based in Leuven, that aims to address the challenges we face today in pediatric drug development (2). EPTRI is dedicated to bridging the gap in pediatric therapeutics by fostering a collaborative, open-science research environment that allows researchers and clinicians to work together without geographical, institutional or financial barriers, to bring new pediatric medicines on the market for the benefit of the health of children. By facilitating an open interdisciplinary collaboration and encouraging policy changes, EPTRI strives to ensure that children have access to safe and effective treatments. Despite recent advances, a significant portion of pediatric drugs remains undeveloped specifically for children, leading to gaps in safety, efficacy, and appropriate dosing. These gaps result in unmet therapeutic needs and limited treatment options for young patients. EPTRI's mission is to address these gaps by fostering research and advocating for supportive policies and funding.

Building on this mission, this Research Topic brings together diverse studies that collectively expand our understanding of pediatric drug development through various perspectives. These eight articles highlight the concerted efforts in pediatric research by:

• Capturing the current state of evidence in pediatric drug development, revealing significant advancements and ongoing challenges. By reviewing the perspectives of healthcare workers on pediatric medicine issues and summarizing the gaps and needs from a practical standpoint (Barbieri et al.), one can recognize the importance of these aspects to improve pediatric healthcare outcomes in the future.
• Expanding our understanding of the methodologies and technologies that can enhance pediatric drug development, such as the application of physiologically-based pharmacokinetic modeling for optimizing gentamicin dosing in neonates and infants (de Hoop-Sommen et al.); or the efficacy of remimazolam and propofol combined with low-dose esketamine for pediatric same-day painless bidirectional endoscopy that highlights the potential for improved anesthetic protocols in pediatric care (Chu et al.); or the investigation of the carbon source utilization characteristics and syntrophic effects of *Bifidobacterium longum* and *Pediococcus pentosaceus* using an *in vitro* intestinal simulation system that provides valuable insights into gut microbiota interactions and their implications for pediatric health (Li et al.).
• Addressing the clinical and real-world evidence critical for pediatric drug development. For example, Wang et al. present a randomized controlled meta-analysis on the impact of different rotavirus vaccines on intussusception incidence, providing essential postmarketing data on vaccine safety and efficacy in pediatric populations. Degraeuwe et al. discuss the development and performance of the c4c national clinical trial networks aimed at optimizing pediatric trial facilitation, emphasizing the importance of robust clinical trial networks in generating reliable data for pediatric drug approvals. In addition, Schluterman et al. provide a mini-review on the relevant physiology and assessment techniques for evaluating drug palatability in young children, highlighting the critical role of palatability in medication adherence, an often-overlooked factor that can significantly impact therapeutic outcomes. Ye et al. examined the evidence of subtherapeutic concentrations in piperacillin/tazobactam treatment in children, emphasizing the need for precise dosing to achieve therapeutic efficacy.

This Research Topic reflects the diversity of disciplines and methodologies that are essential for advancing pediatric drug development. The authors come from various backgrounds, including clinical pharmacology, biomedical engineering, pediatrics, and pharmaceutical sciences. Their contributions encompass a wide range of approaches, from clinical trials and meta-analyses to *in vitro* studies and pharmacokinetic modeling. This diversity is crucial for a comprehensive understanding of pediatric drug development and highlights the importance of interdisciplinary collaboration. While the contributions in this Research Topic mark significant progress, they also highlight areas requiring further investigation, such as the need for more extensive clinical trials involving diverse pediatric populations to ensure that findings are broadly applicable, or the integration of advanced technologies, such as artificial intelligence and machine learning, in modeling and simulation studies.

The studies featured in this Research Topic exemplify the strides being made in pediatric drug development. By bridging gaps between basic research and clinical application, these contributions pave the way for safer, more effective pediatric treatments. The ongoing commitment to addressing the unique needs of children in drug development will ultimately lead to improved health outcomes and quality of life for young patients worldwide.
